# New insights into the functions and regulations of MAP215/MOR1 and katanin, two conserved microtubule-associated proteins in Arabidopsis

**DOI:** 10.1080/15592324.2023.2171360

**Published:** 2023-01-31

**Authors:** Xiayan Liu, Fei Yu

**Affiliations:** a State Key Laboratory of Crop Stress Biology for Arid Areas and College of Life Sciences, Northwest A&F University, Yangling, Shaanxi, China; b Institute of Future Agriculture, Northwest A&F University, Yangling, Shaanxi, China

**Keywords:** Microtubule, microtubule associated-protein (MAP), MOR1/MAP215, katanin, plant cell morphogenesis, plant cell division

## Abstract

Plant microtubules (MTs) form highly dynamic and distinct arrays throughout the cell cycle and are essential for cell and organ morphogenesis. A plethora of microtubule associated-proteins (MAPs), both conserved and plant-specific, ensure the dynamic response of MTs to internal and external cues. The MAP215 family MT polymerase/nucleation factor and the MT severing enzyme katanin are among the most conserved MAPs in eukaryotes. Recent studies have revealed unexpected functional and physical interactions between MICROTUBULE ORGANIZATION 1 (MOR1), the Arabidopsis homolog of MAP215, and KATANIN 1 (KTN1), the catalytic subunit of katanin. In this minireview, we provide a short overview on current understanding of the functions and regulations of MOR1 and katanin in cell morphogenesis and plant growth and development.

Plant microtubule (MT) cytoskeleton plays vital roles in cell division and cell morphogenesis. MTs form distinct types of arrays at different stages of the plant cell cycle. In interphase cells, cortical MT (cMT) arrays are closely associated with the plasma membrane and are generally believed to guide the movement of the cellulose synthase and direct the deposition of cellulose microfibrils.^1,2^ During cell division, three types of MTs arrays, the preprophase band (PPB), the spindle, and the phragmoplast MT arrays are observed in plants.^3^ The PPB is a ring-like structure encircling the nucleus at the preprophase and forecasts the position of the incipient cell division plane.^4^ In plant cell cytokinesis, phragmoplast MT arrays, which are short MT bundles formed on each side of the division plane, facilitate the delivery of vesicles for the formation of the cell plate and the future cell wall.^5^ Notably, the PPB and the phragmoplast are unique to land plants and may represent evolutionary novelties for plant terrestrialization.^6^

The sessile life style of plants necessitates dynamic and robust responses of MT arrays to developmental, hormonal, and environmental signals.^3^ The dynamic properties of MTs are regulated by a plethora of microtubule-associated proteins (MAPs), defined as proteins that directly bind to MTs.^7^ Some highly conserved MAPs, such as the MT polymerase/nucleation factor XMAP215 family protein and the MT severing complex katanin, are present in plants.^8–10^ Moreover, a growing list of plant-specific MAPs, such as the MAP65 family and the IQD family, has been recognized as important regulators of MT dynamics in plants.^7,11–13^ These findings suggest that MT dynamics are governed by both conserved and novel regulatory mechanisms in plants. Although tremendous progress has been made toward the understanding of the function of plant MAPs, our knowledge of the genetic, functional, and physical interaction network of plant MAPs remains limited.

XMAP215 (Xenopus microtubule assembly protein of 215 kD), the founding member of the MAP215 family, was originally purified from Xenopus egg extracts, and XMAP215 homologs have since been identified in many eukaryotic organisms.^14^ In Arabidopsis, *MICROTUBULE ORGANIZATION 1* (*MOR1*) encodes the MAP215 homolog.^8^ MOR1/MAP215 homologs contain tandem repeats of tubulin-binding TOG domains in their N-termini and a less conserved basic C-terminal region.^15^ Functionally, XMAP215 acts as an MT polymerase as it tracks the plus-end of MTs and is able to drastically increase the rate of MT polymerization in vitro and in vivo.^16^ In addition, studies in yeasts, Xenopus, and mammalian cells also showed that XMAP215 homologs participate in MT nucleation.^17–19^ TOG domains are essential for MT nucleation and the MT polymerase activity of XMAP215 while the C-terminus of XMAP215 mediates its interaction with γ-tubulin in the γ-tubulin ring complex.^19^ In Arabidopsis, the C-terminal GFP fusion of a truncated MOR1 containing the first two TOG domains (TOG12-GFP) was able to bind to MTs.^20^ We have recently found that the C-terminal region of MOR1 interacted with the p60 catalytic subunit of the MT severing enzyme katanin.^21^

*MOR1* is an essential gene in Arabidopsis and hypomorphic alleles of *MOR1* have played critical roles in elucidating *MOR1* functions. Initially, viable alleles of *MOR1* were identified in a pioneering genetic screen for mutants with aberrant MT organizations at elevated temperature.^8^ Temperature-sensitive alleles of *MOR1*, including *mor1-1* (L174F) and *mor1-2* (E195K), were indistinguishable from the wild type (WT) at the permissive temperature of 21°C.^8^ However, at 29°C, while WT cells maintained intact cMT arrays, cMTs were fragmented and disorganized in *mor1-1*.^8^ Moreover, aberrant PPB and phragmoplast MT arrays were common and the duration of mitosis was significantly increased in *mor1-1* at 31°C, which led to mispositioned cell plate and cell wall.^22^ These abnormalities in MT arrays at high temperature led to severely stunted growth and left-handed spiral growth in *mor1-1*.^8^ In addition to MT defects, *mor1-1* also showed hypersensitivities to microfilament (MF)-destabilizing drugs latrunculin B and Cytochalasin D, suggesting that MOR1 is involved in the crosstalk between MFs and MTs.^23^ Knockout alleles of *MOR1* were isolated as *gemini pollen1* (*gem1*) mutants based on their severe defects in pollen development.^24,25^ In *gem1* mutants, misguided phragmoplasts in post-meiotic cytokinesis of microspores caused the formation of ectopic cell walls, altered pollen cell fate, and infertility.^25^ An additional hypomorphic allele of *MOR1, root initiation defective 5* (*rid5*) (R96C), was isolated based on genetic screens for mutants with decreased ability to form adventitious roots from hypocotyl explants.^26^ At the restrictive temperature of 28°C, a higher concentration of auxin was needed for the formation of adventitious roots in *rid5* than in the WT, suggesting that MOR1 plays a role in auxin-regulated processes.^26^ Recently, we have identified viable *mor1* alleles, *mor1-10* (E1254K) and *mor1-11* (E224K), through genetic screens for mutants with increased sensitivity to the MT-destabilizing drug propyzamide.^21^ Under propyzamide treatment, the elongation of root cells and the orientation of cell division planes in the root meristem were more severely affected in *mor1-10* than in the WT.^21^ Closer examination of the MT arrays during cell division revealed that propyzamide treatment led to higher frequencies of aberrant PPB, spindle, and phragmoplast MT arrays in *mor1-10* than in the WT.^21^ Interestingly, *mor1-10* showed a left-handed spiral growth pattern and abnormal pavement cell morphology under normal growth conditions, in contrast to *mor1-1* and *mor1-2*.^21^ Taken together, these findings suggest that MOR1 activity is critical for MT organizations, as well as plant cell and organ morphogenesis.

Understanding the dynamic MT localization patterns of MAPs provides invaluable information on MAP functions. Immunofluorescence studies of MOR1 localizations using different MOR1 antibodies yielded important, albeit inconsistent findings on the dynamics of MOR1 localization, and the lack of functional MOR1-GFP fusion in planta has long hampered the elucidation of MOR1 function.^22,25^ The recently reported functional MOR1-GFP fusion revealed that MOR1-GFP predominantly tracked the plus-ends of cMTs, with also a weaker association with the cMT lattice in interphase cells (Figure 1a).^21^ In addition, MOR1-GFP localized to the nucleation site prior to MT branching and subsequently tracked the plus-ends of emerging new MTs (Figure 1a).^21^ In hypomorphic *mor1-10* mutants, compromised plus-end dynamics including slower growth rate and longer pause periods were observed under both control and propyzamide treatment, supporting a role for MOR1 in regulating cMT plus-end dynamics.^21^ Consistently, plus-end dynamics and the association of EB1 at cMT plus-ends were also reduced in *mor1-1* at the restrictive temperature of 31°C.^27^ Intriguingly, in dividing cells, MOR1-GFP signals were distributed relatively evenly on the PPB, the spindle, and the phragmoplast MT arrays, in contrast to the preferential plus-end localization pattern on cMTs (Figure 1b-1d).^21^ These findings suggest distinct regulatory mechanisms controlling the MT localization patterns of MOR1 in interphase and dividing cells.

**Figure 1.**
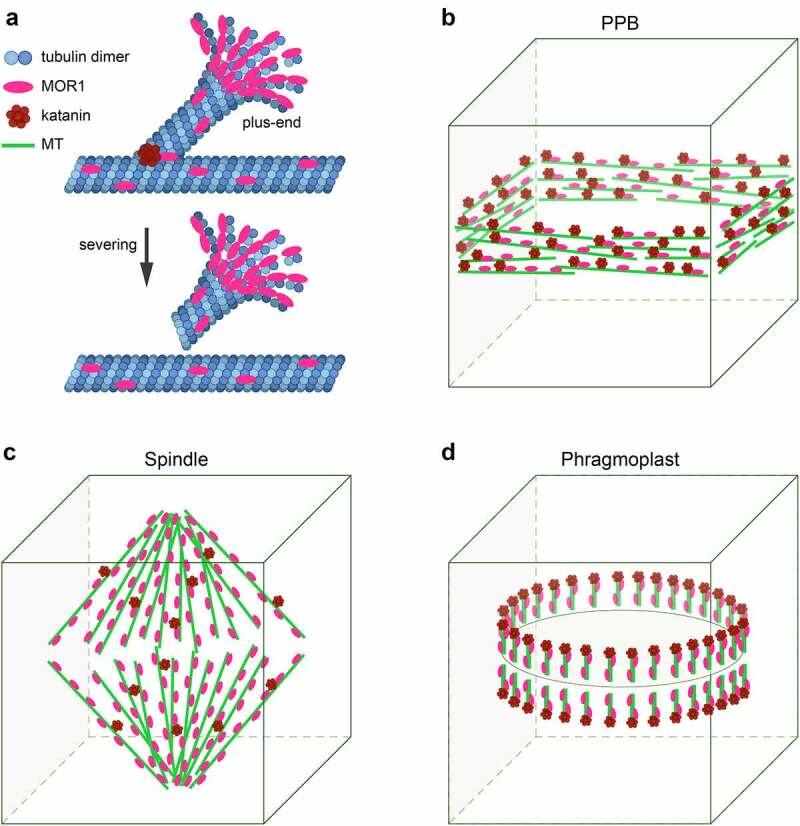
**Dynamic localizations of MOR1 and KTN1 at different stages of plant cell cycle**.^21^

A, In interphase cells, MOR1 is enriched at plus-ends of cMTs and also binds to cMT lattice. MOR1 facilitates the recruitment of KTN1 to cMT crossover/branching sites where cMTs are severed by katanin. B-D, During cell division, MOR1 and KTN1 are required for the organization of PPB and phragmoplast MT arrays. MOR1 is evenly distributed on PPB, spindle, and phragmoplast MT arrays. KTN1 is prominently associated with PPB and enriched at the distal ends of phragmoplast MTs while low abundance of KTN1 is found on spindle MTs.

MT severing enzymes are another important category of MAPs that modulate the MT dynamics through disassembling MTs using energy from ATP hydrolysis.^28^ Initially purified from sea urchin eggs, katanin is the first discovered MT-severing enzyme, composed of the p60 catalytic subunit and the p80 regulatory subunit.^29^ The p60 subunit, containing an AAA-type ATPase domain in the C-terminus and an MT-interacting and trafficking (MIT) domain in the N-terminus, is able to sever MTs in vitro and its ATPase activity is stimulated by MTs.^30,31^ The p80 subunit has an N-terminal WD40 domain and a C-terminal conserved p80 domain (p80-CTD) and promotes the centrosome localization and MT-binding of p60 in animal cells.^29^ Structural analyses using X-ray crystallography and cryo-EM revealed that the katanin complex is a hexamer which cycles between open spiral and closed ring conformations depending on the ATP-binding state.^32^ Moreover, the interaction between katanin subunits was mediated by p80-CTD and p60-MIT.^33^ The p60-MIT:p80-CTD complex can also assemble into heterotetramers, and this heterotetramer form of katanin affected its MT-binding preference, suggesting that katanin complexes may exist in different oligomeric forms with distinct functional properties.^33^

Homologs of katanin subunits have also been identified in plants.^34^ In Arabidopsis, a single copy gene, *KATANIN 1* (*KTN1*) encodes the p60 subunit while four homologous genes (*KTN80.1, KTN80.2, KTN80.3*, and *KTN80.4*) code for the p80 subunit.^10,12^ Live imaging of cMTs has revealed that severing occurs mostly at the MT branching and crossover sites in Arabidopsis.^35,36^ Dual-imaging of GFP-KTN1 and mRFP-labeled cMTs revealed that KTN1 is recruited to cMT crossover and branching sites (Figure 1a).^35,36^ The likelihood of cMT severing is influenced by the stability of the cMT crossover and the dwelling time of KTN1 at the crossover/branching site.^35^ KTN1 has distinct and dynamic MT localization patterns during cell division.^21,37^ In dividing cells, GFP-KTN1 signals are evenly distributed on PPB MTs, but are specifically enriched at the distal ends (minus-ends) of phragmoplast MTs (Figures 1b and 1d).^21,37^ Compared with the prominent signals of GFP-KTN1 on PPB and phragmoplast MTs, spindle-localized GFP-KTN1 signals are minimal.^21,37^ However, considering the spindle defects found in *ktn1* mutants, it is possible that a small amount of KTN1 may also associate with spindle MTs (Figure 1c).^38^

MT severing serves as a means to generate new MTs and is necessary for the reorientation of cMT arrays in response to environmental signals.^39,40^ It is also well established that katanin-mediated severing is required for the release of minus-ends of daughter MTs from the branching sites, where new MTs are nucleated in plant cells.^35,36^ The development of super-resolution imaging protocols for plant MTs also helped to offer more details on the role of KTN1 in MT organization in both interphase and dividing cells.^38,41–43^ In Arabidopsis, loss-of-function mutants of *KTN1* showed abolished severing of cMTs, increased occurrence of incomplete PPBs, abnormalities in the movement of the spindle and the phragmoplast, longer durations of mitosis and cytokinesis, and defects in cell plate formation.^21,35–38,44^ At the cellular and organismal level, mutants of *KTN1* showed a spectrum of developmental defects associated with compromised MT organization, cell elongation, and cell division, including small plant statues, altered pavement cell, trichome and conical cell morphology, cell wall and root hair defects, misguided pollen tube growth, reduced fertility and seed set, and abnormal stomatal patterning.^9,10,45–54^ Robust responses of plants to hormonal and environmental cues also required KTN1.^39,55–59^ For example, blue light-induced reorganization of cMT arrays and phototropic growth were found to be defective in *ktn1* mutants.^39^ Despite the critical roles of katanin, recent findings also suggested a katanin-independent MT array reorganization mechanism in Arabidopsis.^60^ When Arabidopsis seedlings were germinated on agar surface, KTN1 was required for reorganizing cMTs to form the apical hook.^60^ However, hook formation defects in *ktn1* mutant can be rescued when seedlings were germinated within soil and subjected to external mechanical constraints, suggesting that mechanical force can drive MT array reorganization in a katanin-independent manner.^60^ Arabidopsis p80 is required for recruiting p60 to the cMT crossover/branching sites, and the disruption of all four p80 coding genes in Arabidopsis led to mutant phenotypes that were highly reminiscent of *ktn1* mutants, indicating that both subunits are indispensable for the severing activity of the katanin complex.^12^

Given the critical functions of katanin, it is not surprising that the severing activity of katanin is intimately regulated in plants. SPIRAL2 (SPR2), a plant-specific MAP, has been shown to accumulate at cMT crossover sites and prevent KTN1-mediated severing in Arabidopsis pavement cells.^61^ In contrast, a reduced severing frequency was reported in hypocotyl cells in *spr2-2* mutants, suggesting that SPR2 is a positive regulator of cMT severing.^62^ It has been suggested that SPR2 can localize to and stabilize cMT minus-ends, thus increasing the lifetime of crossovers and the likelihood of severing at crossovers.^62^ Plus-end dynamics also regulates cMT severing. The localization of CLIP-Associating Protein (CLASP), a well-conserved MAP, to the newly generated cMT plus-ends after severing prevented the shrinkage of plus-ends, which facilitated the amplification and reorientation of cMT arrays through katanin-mediated severing.^63^ The recruitment of katanin to cMT branching sites, but not crossover sites, required homologs of a conserved centrosomal MT-anchoring complex Msd1-Wdr8 in Arabidopsis.^64^ In *wdr8* mutants, branched cMTs were observed to detach from the branching sites in the absence of katanin and the loss of Msd1 or Wdr8 could partially rescue the cell elongation defects of *ktn1* in *msd1a msd1b ktn1* triple mutants and *wdr8 ktn1* double mutants, respectively.^64^ The augmin complex has been reported to antagonize katanin-mediated cMT severing in pavement cells.^65^ Localization of augmin to cMT crossover sites stabilized these sites and prevented the severing by katanin.^65^ Moreover, KTN1 interacts directly with multiple factors to coordinate cMT severing and the formation of highly ordered transverse parallel MT arrays. In pavement cell morphogenesis, KTN1 was required for Rho-GTPase ROP6 and its effector RIC1 to promote the formation of highly ordered parallel cMT arrays in the neck region.^49^ Mechanistically, RIC1 directly interacted with KTN1 and promoted the severing activity of KTN1.^49^ In an Arabidopsis gain-of-function mutant *abnormal shoot6-1D* (*abs6-1D*), an increased cMT severing frequency correlated with highly ordered transverse parallel cMT arrays in pavement cells, highly elongated pavement cells, and an overall elongated plant organ shape.^53^ *ABS6* encodes a member of the IQ67 domain (IQD) family MAP, IQD16, that directly interacted with KTN1 and promoted the recruitment of KTN1 to the cMT crossover/branching sites.^53^ Another member of the Arabidopsis IQD family, IQD21, was also reported to interact with KTN1 and promote KTN1-mediated cMT severing to modulate the formation of pavement cell indentations.^66^ During cytokinesis, the enrichment of KTN1 at the distal ends of the phragmoplast required CORD4 and CORD5, members of a plant-specific MAP family, and abnormally long and oblique phragmoplast MTs and delayed centrifugal expansion of phragmoplasts were observed in *cord4 cord5* double mutants.^37^ Recently, KTN1 has been shown to interact with subunits of PP2A, a conserved protein phosphatase complex, and the dephosphorylation of KTN1 by PP2A stabilized KTN1.^67^ Unexpectedly, a direct interaction between KTN1 and the carboxyl region of MOR1 was reported.^21^ Consistently, double mutants of *MOR1* and *KTN1* showed enhanced defects in anisotropic cell expansion and the orientation of cell division planes, supporting a functional link between *MOR1* and *KTN1*.^21,68^ Interestingly, while MOR1-mediated plus-end dynamics was not affected in *ktn1* mutants, reduced severing frequency and delayed recruitment of KTN1 to the cMT crossover/branching sites were observed in *mor1-10*, indicating that MOR1 is a positive regulator of KTN1-mediated cMT severing.^21^

With the tremendous progress in plant MT research, we are beginning to appreciate the intricate functional network of plant MAPs in regulating MT dynamics to ensure developmental plasticity and robust response to the ever-changing environment. On one hand, consider the significant differences between MT organizations in plant and animal cells, it would not be surprising to discover unexpected functional interactions between conserved MAPs in plants. On the other hand, the expanding complement of plant-specific MAPs provides novel regulatory components to MT regulatory networks. Future studies on the genetic, physical, and functional interactions of plant MAPs will likely uncover new paradigms in the regulation of plant MT cytoskeleton.
